# Assessment of health-related quality of life among patients with obesity, hypertension and type 2 diabetes mellitus and its relationship with multimorbidity

**DOI:** 10.1371/journal.pone.0289502

**Published:** 2023-08-04

**Authors:** Shahid Shah, Ghulam Abbas, Ayesha Aslam, Fawad Ahmad Randhawa, Faiz Ullah Khan, Haris Khurram, Usman Rashid Chand, Muhammad Hammad Butt, Tauqeer Hussain Mallhi, Yusra Habib Khan

**Affiliations:** 1 Faculty of Pharmaceutical Sciences, Department of Pharmacy Practice, Government College University Faisalabad, Faisalabad, Pakistan; 2 Faculty of Pharmaceutical Sciences, Department of Pharmaceutics, Government College University Faisalabad, Faisalabad, Pakistan; 3 Department of Neurology, King Edward Medical University, Lahore, Pakistan; 4 Department of Endocrinology, King Edward Medical University, Lahore, Pakistan; 5 Department of Pharmacy Administration and Clinical Pharmacy, School of Pharmacy, Xi’an Jiaotong University, Xi’an, China; 6 National University of Computer and Emerging Sciences, Chiniot-Faisalabad Campus, Chiniot, Pakistan; 7 Faculty of Pharmacy, Department of Medicinal Chemistry, Uppsala University, Uppsala, Sweden; 8 Department of Clinical Pharmacy, College of Pharmacy, Jouf University, Sakaka, Saudi Arabia; Faculty of Health Sciences - Universidade da Beira Interior, PORTUGAL

## Abstract

Obesity, hypertension (HTN) and type 2 diabetes (T2D) are among the multifactorial disorders that occur at higher prevalence in a population. This study aims to assess the health-related quality of life (HRQoL) of patients with obesity, HTN and T2D individually and in the form of multimorbidity. A questionnaire-based cross-sectional study was conducted among the patients in 15 private clinics of Punjab, Pakistan. A stratified random sampling technique was used to collect the data from patients with obesity, HTN and T2D or their comorbidity. A total of 1350 patients responded by completing the questionnaire. The HRQoL of these patients was assessed using the EQ-5D-5L questionnaire (a standardized instrument for measuring generic health status). Statistical analysis was performed using chi-square test, Mann-Whitney U test, and Kruskal-Wallis test. Multivariate linear regression model was used to model the visual analogue scale (VAS) score. In total, 15% of patients had combined obesity, HTN and T2D; 16.5% had HTN and T2D; 13.5% had obesity and HTN and 12.8% had obesity and T2D. Only 15.8% of patients had obesity, 14.3% had HTN, and 12% had T2D. Mann Whitney-U test gave the statistically significant (*p = <0*.*001*) HRQoL VAS score55.1 (±23.2) of patients with the obesity. HRQoL VAS scores of patients with obesity were found to be higher when compared to patients with both T2D 49.8 (±15.4) and HTN 48.2 (±21). Diagnosis of one, two and three diseases showed significant results in VAS with all variables including gender (*p = 0*.*004*), educational level (*p = <0*.*001*), marital status (*p<0*.*001*), residence (*p = <0*.*001*), financial situation (*p = <0*.*001*) and monthly income (*p = <0*.*001*). The most frequently observed extremely problematic dimension was anxiety/ depression (47%) *and the self-care (10%) was the least affected*. Patient HRQoL is decreased by T2D, HTN, and obesity. The impact of these diseases coexisting is more detrimental to HRQoL.

## Introduction

Nearly all populations experience obesity, hypertension (HTN), and type 2 diabetes (T2D) [1]. According to the World Health Organization (WHO), obesity, HTN, and T2D are three of the top five persistent risk factors for cardiovascular mortality worldwide [2]. Recent studies have identified obesity and overweight as risk factors for several diseases, including T2D and HTN [3]. Numerous epidemiologic studies show prevalence rates of obesity, HTN, and diabetes separately in the general population or in individuals with diabetes, but frequently do not differentiate between type 1 and type 2 diabetes [4]. Obesity and insulin resistance are the most important factors in the relationship between metabolic syndrome and oxidative stress and should therefore be quickly identified and treated [5]. Despite methodological differences, obesity showed significant, potentially plausible association with HTN and T2D [6].

Obesity, HTN, and T2D are chronic conditions that often coexist and are associated with significant morbidity and mortality worldwide, including in Pakistan [7]. Deaths from obesity, HTN and T2D are highest in low- and middle-income countries and lowest in high-income countries [8]. The most impacted are the poorest citizens of every nation. Furthermore, in terms of morbidity, mortality, and healthcare expenses, obesity, HTN, and T2D pose a significant burden [9]. In general, burden of disease study of obesity, HTN, and T2D have societal impact in terms of cost of illness [10–12]. These conditions could lead to physical limitations, pain, psychological distress, reduced social functioning, and decreased overall well-being. The presence of multiple chronic conditions, or multimorbidity, could further compound the negative impact on *Health-related quality of life* (HRQoL). HRQoL is the extent to which one’s usual or expected physical, emotional, and social well-being are affected by a medical condition or its treatment [13]. A study carried out in Pakistan revealed that patients with HTN had low HRQoL [14]. Another study focused on HRQoL in T2D patients and concluded that those patients’ HRQoL was impaired [15]. One of the primary issues that the healthcare and social care systems are currently confronting is how to assist individuals who are living with chronic conditions like obesity, HTN, and T2D to maintain an HRQoL [16]. It is important to consider how multimorbidity affects a person’s HRQoL and personal priorities while managing patients with multimorbidity [17]. The impact of multimorbidity on HRQoL in Pakistan is not well understood, yet. To measure the impacts of these three diseases on a more global level, however, information on health-related quality of life (HRQoL) should be obtained. Our goal was to assess the HRQoL in both an individual and multimorbid setting for individuals with obesity, HTN, and T2D.

## Methods

### Study design and subjects

A questionnaire based cross-sectional multicenter study over a period of 3 months from June to August 2022. A stratified random sampling technique was adopted to collect the data from the patients with obesity, HTN and T2D individually and in the form of multimorbidity with the help of physicians. First, we selected 5 large populated cities (Gujranwala, Rawalpindi, Lahore, Multan and Faisalabad) in Punjab, Pakistan, and then randomly selected 3 private clinics in each city and received 266 responses from each city (each city responded with an average of 80–90%). Thus, we contacted 1580 patients. Of these, 230 patients refused to participate, and 20 incomplete questionnaires were not included. Items included in the questionnaire cover all the aspects of the relationship between HTN, T2D, obesity and HRQoL and each item was linked to the objectives of this study as well.

Mobility, self-care, usual activities, pain/discomfort, and anxiety/depression were the five characteristics of health that the EQ-5D-5L was used to describe and value. After being reviewed by six experts—two in cardiology, two in endocrinology, and two in pharmacy education—the questionnaire was distributed to the participants. 35 patients received the questionnaire as part of a pilot study in order to identify errors and misunderstood questions. These participants’ responses were left out of the final analysis. After explaining the purpose of the study to the participants, they were given the EQ-5D-5L questionnaire to complete. After explaining the study’s protocols to non-educated participants, an interview was done with their written and verbal consent.

### Patient and public involvement

The design, analysis, and interpretation of this study were done without the involvement of either patients or public, and neither group will be involved in the results’ dissemination.

### Ethics approval

Government College University Faisalabad’s Institutional Review Board gave its approval (GCUF/ERC/3208) to the study’s design, which complies with the Helsinki Declaration. After obtaining an informed consent form from each participant, a private interview was conducted with them to complete the questionnaires.

### Inclusion criteria

Age 40–66 years, a BMI greater than 25 kg/m^2^, and, for those with T2D, a HbA1c level between 57 and 78 mmol/mol [International Federation of Clinical Chemistry (IFCC) standard], which is comparable to 7.4–9.3% [National Glycohemoglobin Standardization Program (NGSP) standard], were the inclusion criteria. Aging is a strong risk factor for many chronic diseases [18]. Patients who met the exclusion criteria for the study were those who were physically disabled, mentally ill, or who used drugs of abuse such Piper *betle* (pan), *Dalbergio sisso* (sheesha), or Areca *catechu* (ghuttka).

### Quality of life instrument

The HRQoL of obesity, HTN, and T2D was assessed using the EQ-5D-5L instrument. Due to the EQ-5D’s comprehensive and simple features, the major goal of employing it was to ensure high response rates. Furthermore, the earlier investigations have demonstrated the validity and reliability of the method. Previous studies has shown a strong association between the dimensions of this instrument and those of other commonly used instruments [19].

By completing the registration form on the EuroQol website, consent was obtained for the use of the necessary version of the EQ-5D-5L. Two pages make up the EQ-5D-5L: a description page and a visual analogue scale (VAS). The five aspects of health that make up the description page are mobility, self-care, usual activities, pain or discomfort, and anxiety or depression. There are 3,125 different possible states of health, with each defined by one of five levels of each dimension: no problem, slight problems, moderate problems, serious problems and extreme problems.

By employing VAS, the current condition of health was directly assessed. The VAS is essentially a 20 cm long graduated scale with numbers ranging from 0 to 100. The range from 0 to 100 denotes the range of possible health states, with 100 denoting the best conceivable health state. The respondents were asked to mark on a scale, and the resultant value was used as a quantitative indicator of the respondents’ perceived health outcomes. The information on these two pages helped us determine a person’s health state. The questionnaire was completed by the patients in accordance with their current circumstances, and a five-digit number was obtained and used to represent that person’s current state of health.

### Statistical analysis

Data were analyzed by using Statistical Package for Social Sciences (IBM SPSS Statistics for Windows, version 21.0, Armonk, NY: IBM Corp.). Descriptive statistics were used to describe the study findings. Mean ± standard deviation (SD) and frequency (%) were used to describe continuous and categorical variables respectively. Chi-square test is used to measure bivariate association. Mann Whitney U test and Kruskal Wallis test were used to test the effect of different factors on VAS score. Multivariate Regression was used to evaluate the significance of different factors and variables on VAS score for different diseases. A *p-value* of less than 0.05 was considered statistically significant.

## Result

### Sample characteristics

**Fig 1:**
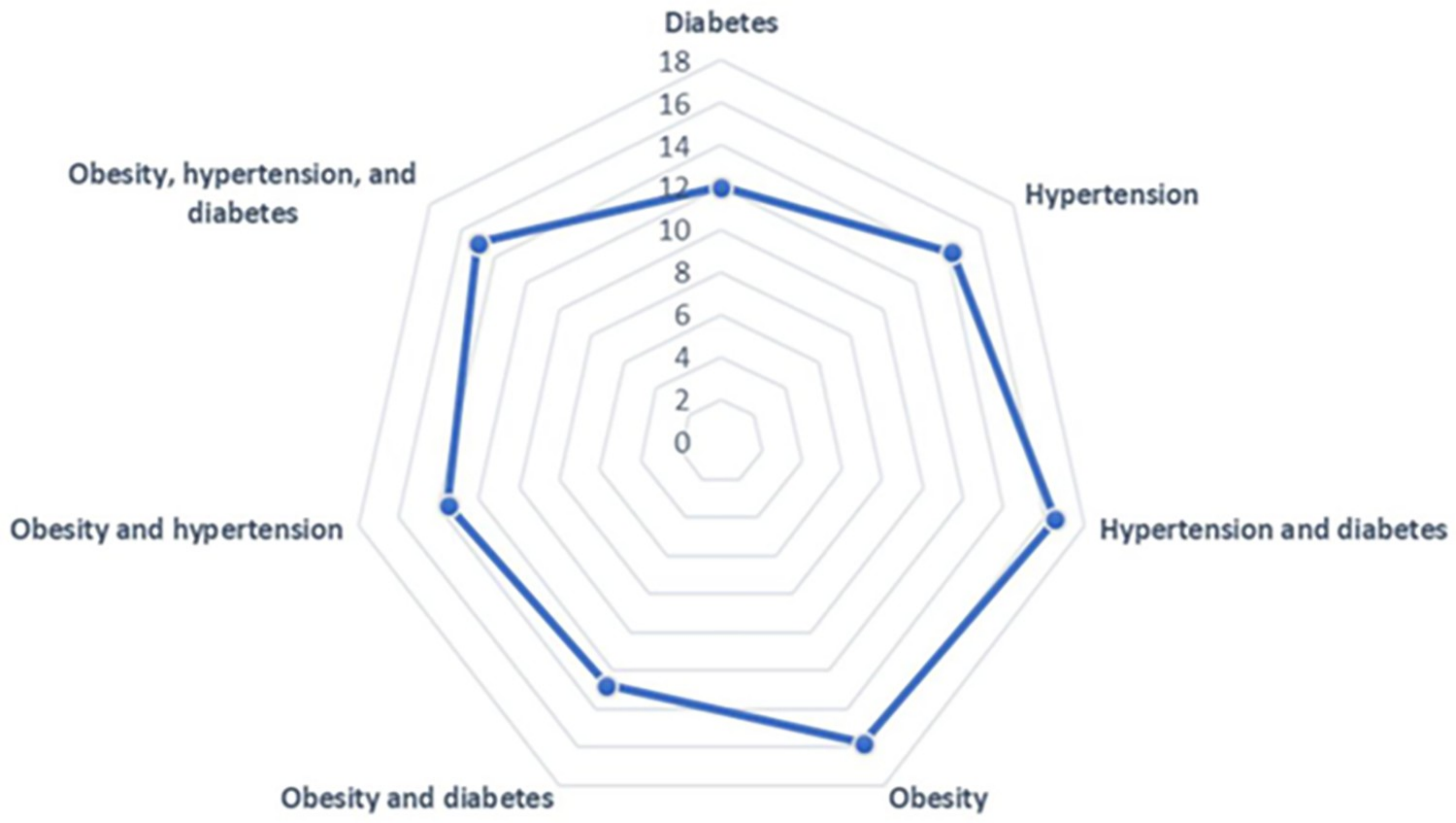
Percentage of cases where diabetes, hypertension, and obesity are diagnosed both separately and together.

A total of 1330 patients completed EQ-5D questionnaire. In total, 15% of patients had combined obesity, HTN and T2D; 16.5% had HTN and T2D; 13.5% had obesity and HTN and 12.8% had obesity and T2D. Only 15.8% of patients had obesity, 14.3% had HTN, and 12% had T2D as shown in Fig 1. Patients’ socio-demographic characteristics are presented in Table 1. Demographic data established that, out of the total study population, 60.5% were male. Furthermore, 59% of patients were higher school graduates, 63% were married, 64.5% were living in rural areas, 72% of them were working, 51.3% had medium level financial satisfaction, and 42% had an income level 30 to 60 thousand Pakistani rupees. Statistically significant correlation was found between socio demographic characteristics and diseases like HTN, T2D and obesity. In comparison to male, females are more likely (55%) to have several morbidities. Similarly, married people have more diseases than single ones. Patients with higher studies had 34.2% HTN and 59.2% obesity. 54.4% of patients with HTN, 68% of those with T2D, and 72.4% of obese patients were employed. Obesity patients make up 51.3% of those with a medium level of financial happiness; in contrast, those who said they were financially satisfied had 53.2% HTN and 73.3% T2D. 42.1% of obese patients, 45.6% of HTN patients, and 44% of T2D patients reported monthly incomes between 30,000 and 60,000.

**Table 1:** Frequency distribution and association measures of demographic: Obesity HTN or T2D.

Variables	Categories	Obesity	*p-value*	HTN	*p-value*	T2D	*p-value*
		Yes		Yes		Yes	
Gender	Female	300 (39.5%)	0.004	300 (38%)	<0.001	470 (62.7%)	<0.001
	Male	460 (60.5%)		490 (62%)		280 (37.3%)	
Marital status	Married	480 (63.2%)	<0.001	630 (79.7%)	<0.001	720 (96%)	<0.001
	Unmarried	280 (36.8%)		160 (20.3%)		30 (4%)	
Educational level	Higher studies	450 (59.2%)	<0.001	270 (34.2%)	<0.001	250 (33.3%)	<0.001
	Secondary education	120 (15.8%)		260 (32.9%)		180 (24%)	
	Primary education	190 (25%)		260 (32.9%)		320 (42.7%)	
Place of residence	Rural	490 (64.5%)	<0.001	380 (48.1%)	<0.001	400 (53.3%)	<0.001
	Urban	270 (35.5%)		410 (51.9%)		350 (46.7%)	
Occupational activity	Not working	210 (27.6%)	<0.001	360 (45.6%)	<0.001	240 (32%)	<0.001
	Working	550 (72.4%)		430 (54.4%)		510 (68%)	
Financial situation satisfaction	Medium	390 (51.3%)	<0.001	340 (43%)	<0.001	190 (25.3%)	<0.001
	No	0 (0%)		30 (3.8%)		10 (1.3%)	
	Yes	370 (48.7%)		420 (53.2%)		550 (73.3%)	
Monthly Income	>100,000	60 (7.9%)	<0.001	60 (7.6%)	<0.001	40 (5.3%)	<0.001
	10,000–30,000	120 (15.8%)		210 (26.6%)		140 (18.7%)	
	30,000–60,0	320 (42.1%)		360 (45.6%)		330 (44%)	
	60,000–90,0	260 (34.2%)		160 (20.3%)		240 (32%)	

### Clinical characteristics

Tables 2 and 3 elaborates on the patients’ clinical characteristics and health. Nearly 93% of respondents who had obesity smoked, and more than 80% of those who had HTN and T2D smoked as well. However, among individuals with HTN (39.2%), ACE inhibitors were the most commonly used drugs. Obese, HTN and T2D patients all involved in some form of physical exercise during their leisure time, with respective percentages of 59.2%, 55.7% and 81.3%. About 68% of T2D patients simply took tablets, whereas 25.3% received insulin treatment in addition to their medication. 45–55% had a familial history of obesity, 47–60% had a history of T2D, and 23–60% reported having HTN. Obesity, HTN, and T2D were all recognized as illnesses by 72.4%, 68.4%, and 72% of those who had them, respectively. A very low percentage of patients 18.4% (Obese), 36.7% (HTN) and 20% (T2D) were hospitalized. 1 to 5 years of disease duration reported in 38.2%, 44.3% and 40% of patients with obesity, HTN and T2D respectively.

**Table 2:** Frequency distribution and association measures of clinical characteristics and drug factors of the patient.

Variables	Categories	Obesity	*p-value*	HTN	*p-value*	T2D	*p-value*
		Yes		Yes		Yes	
Are you a smoker?	Yes	710 (93.4%)	<0.001	640 (81%)	<0.001	650 (86.7%)	0.49
	No	50 (6.6%)		150 (19%)		100 (13.3%)	
Medications for T2D	Diet only	200 (26.3%)	<0.001	140 (17.7%)	<0.001	0 (0%)	<0.001
	Insulin	10 (1.3%)		10 (1.3%)		50 (6.7%)	
	Tablets + insulin	60 (7.9%)		150 (19%)		190 (25.3%)	
	Tablets only	490 (64.5%)		490 (62%)		510 (68%)	
Medications for HTN	Adrenergic blockers	80 (10.5%)	<0.001	120 (15.2%)	<0.001	90 (12%)	<0.001
	Angiotensin-converting enzyme	20 (2.6%)		20 (2.5%)		0 (0%)	
	*ACEI/ ARB	310 (40.8%)		310 (39.2%)		330 (44%)	
	*CCB	270 (35.5%)		270 (34.2%)		230 (30.7%)	
	Diuretics	80 (10.5%)		70 (8.9%)		100 (13.3%)	
Dose frequency/ day	Once daily	360 (47.4%)	<0.001	350 (44.3%)	0.006	140 (18.7%)	<0.001
	Thrice	40 (5.3%)		50 (6.3%)		70 (9.3%)	
	Twice a day	360 (47.4%)		390 (49.4%)		540 (72%)	
Any side-effects with the medication	Moderate	210 (27.6%)	0.006	250 (31.6%)	<0.001	270 (36%)	<0.001
	No	530 (69.7%)		500 (63.3%)		460 (61.3%)	
	Severe	20 (2.6%)		40 (5.1%)		20 (2.7%)	

*ACEI: Angiotensin converting enzyme inhibitors, ARB: Angiotensin receptor blockers, CCB: Calcium channel blockers.

**Table 3:** Frequency distribution and association measures of medical condition of the patients, Diagnosis: Obesity HTN or T2D.

Variables	Categories	Obesity	*p-value*	HTN	*p-value*	T2D	*p-value*
		Yes		Yes		Yes	
Any family history of obesity?	No	420 (55.3%)	<0.001	610 (77.2%)	<0.001	400 (53.3%)	<0.001
	Yes	340 (44.7%)		180 (22.8%)		350 (46.7%)	
Any family history of T2D?	No	360 (47.4%)	<0.001	250 (31.6%)	0.001	160 (21.3%)	<0.001
	Yes	400 (52.6%)		540 (68.4%)		590 (78.7%)	
Any family history of HTN?	No	340 (44.7%)	0.33	320 (40.5%)	0.006	300 (40%)	0.003
	Yes	420 (55.3%)		470 (59.5%)		450 (60%)	
Ability to recognize symptoms of disease?	No	210 (27.6%)	0.59	250 (31.6%)	<0.001	210 (28%)	0.38
	Yes	550 (72.4%)		540 (68.4%)		540 (72%)	
Do you hospitalize due to T2D or HTN?	No	620 (81.6%)	<0.001	500 (63.3%)	<0.001	600 (80%)	0.001
	Yes	140 (18.4%)		290 (36.7%)		150 (20%)	
Disease Duration	1 year	210 (27.6%)	<0.001	120 (15.2%)	<0.001	70 (9.3%)	<0.001
	1–5 years	290 (38.2%)		350 (44.3%)		300 (40%)	
	5–10 years	200 (26.3%)		240 (30.4%)		300 (40%)	
	> 10 years	60 (7.9%)		80 (10.1%)		80 (10.7%)	
Blood sugar measurement	2–3 times a day	100 (13.2%)	<0.001	30 (3.8%)	<0.001	50 (6.7%)	<0.001
	Once a day	60 (7.9%)		210 (26.6%)		230 (30.7%)	
	Once a week	170 (22.4%)		200 (25.3%)		200 (26.7%)	
	When feeling bad	430 (56.6%)		350 (44.3%)		270 (36%)	
Blood pressure measurement	2–3 times a day	120 (15.8%)	<0.001	90 (11.4%)	<0.001	40 (5.3%)	<0.001
	Once a day	110 (14.5%)		260 (32.9%)		220 (29.3%)	
	Once a week	170 (22.4%)		200 (25.3%)		130 (17.3%)	
	When feeling bad	360 (47.4%)		240 (30.4%)		360 (48%)	
Stage of HTN	Elevated	140 (18.4%)	0.001	30 (3.8%)	<0.001	130 (17.3%)	<0.001
	Stage 1	310 (40.8%)		350 (44.3%)		310 (41.3%)	
	Stage 2	280 (36.8%)		350 (44.3%)		300 (40%)	
	Stage 3	30 (3.9%)		60 (7.6%)		10 (1.3%)	

### T2D, HTN, obesity in relation to HRQoL VAS score

**Fig 2:**
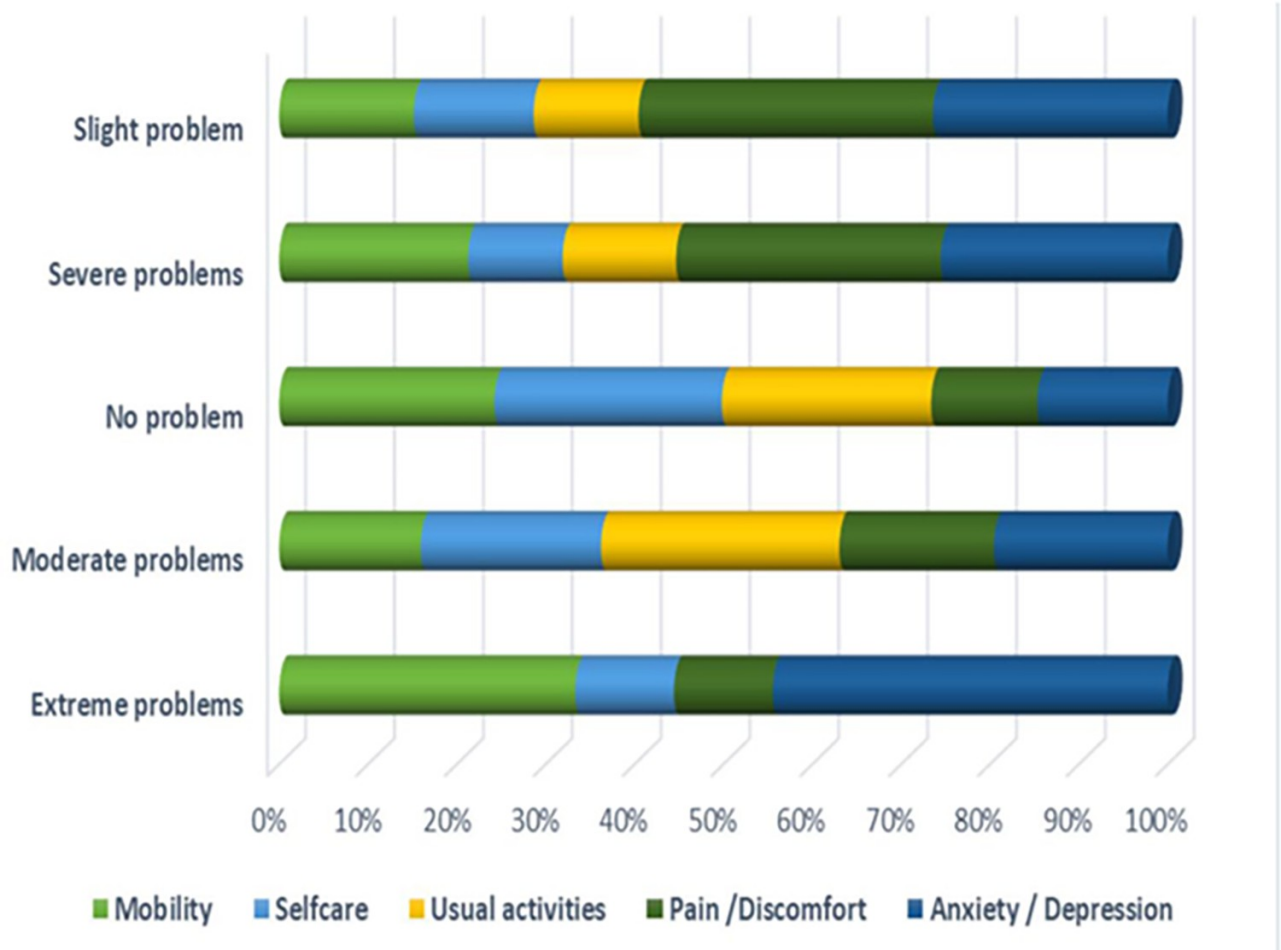
Percentages of individuals who reported the severity according to the EQ-5D-5L dimensions and levels. Mobility, self-care, usual activity, pain/discomfort and anxiety/depression.

HRQoL VAS score of patients with the obesity were 55.1 (±23.2). HRQoL VAS scores of patients with obesity were found to be higher when compared to patients with both T2D 49.8 (±15.4) and HTN 48.2 (±21). It was found that the patients with HTN had the lowest VAS score value as shown in Table 4. Diagnosis of one, two and three disease showed significant results in VAS with all variables including gender, educational level, marital status, residence, occupational activity, financial situation and monthly income as shown in Table 5. Fig 2 displays percentages of participants according to EQ-5D-5L dimensions and levels indicating the degree of severity. Each of the dimensions—mobility, self-care, casual activity, pain/discomfort, and anxiety/depression—was represented by one of five levels, including "no problem," "slight problems," "moderate problems," "severe problems," and "extreme problems." Anxiety/depression was the extreme troublesome component that was most frequently observed (47%).

**Table 4:** Descriptive analysis of VAS for clinical characteristics, medical condition and drug factors.

Variables		Mean (SD)	Median (Q3-Q1)	*p-value*
Diagnose	One Disease	48.5 (29.9)	50 (75–21.25)	<0.001
	Two Diseases	46.8 (14.5)	50 (55–45)	
	Three Diseases	61.3 (14.5)	57.5 (74.25–46.25)	
Obesity	No	42.5 (20.3)	46 (55–30)	<0.001
	Yes	55.1 (23.2)	50 (75–45)	
HTN	No	51.9 (25.3)	50 (75–45)	0.144
	Yes	48.2 (21)	50 (65–35)	
T2D	No	49.6 (29.9)	50 (75–20)	0.492
	Yes	49.8 (15.4)	50 (55–45)	
Any other chronic disease?	No	50.4 (21.9)	50 (65–40)	0.069
	Yes	44.5 (28.6)	47 (70–9.5)	
Are you a smoker?	No	50.2 (23.2)	50 (66.5–36.25)	0.83
	Yes	46.6 (20.1)	50 (55–45)	
Any family history of obesity?	No	46.6 (23.9)	46 (55–30)	<0.001
	Yes	55.2 (19.7)	50 (75–45)	
Any family history of T2D?	No	53.1 (23.6)	45 (67–43)	0.958
	Yes	47.9 (22.3)	50 (65–35)	
Any family history of HTN?	No	49.3 (24.7)	45 (60–30)	0.067
	Yes	50 (21.3)	50 (70–43)	
Any knowledge about disease?	No	62.2 (21.2)	55 (71.5–45)	<0.001
	Yes	45.8 (21.9)	50 (55–30)	
Ability to recognize symptoms of disease?	No	59.4 (26.2)	57.5 (74.25–45)	<0.001
	Yes	46.1 (20.4)	50 (55–35)	
Do you hospitalize due to T2D or HTN?	No	48.8 (22.4)	50 (55–35)	0.001
	Yes	52.6 (24.1)	55 (70–40)	
Is your blood sugar level normal?	No	46.5 (19.5)	45 (58.75–35)	<0.001
	Yes	52.1 (24.8)	50 (70–40)	
Is your blood pressure controlled?	No	51.9 (17.1)	50 (65–45)	0.003
	Yes	48.3 (26)	50 (70–28)	
Disease duration	1 year	54.7 (29.3)	50 (75–28)	<0.001
	1–5 years	48.1 (18.8)	50 (55–40)	
	5–10 years	44.5 (20)	45 (55–41)	
	> 10 years	65.6 (13.6)	62.5 (82.5–51.25)	
Blood sugar measurement	Once a day	51.3 (15)	50 (65–35)	<0.001
	2–3 times a day	68.4 (18.4)	75 (75–75)	
	Once a week	43.4 (15.6)	45 (55–37)	
	When free	48.8 (26.7)	50 (65–28)	
Blood pressure measurement	Once a day	49.6 (16.1)	50 (65–35)	<0.001
	2–3 times a day	64.4 (12.8)	67.5 (75–55)	
	Once a week	42.9 (18.3)	45 (50–43.25)	
	When free	49.1 (27.1)	50 (65–25)	
Stage of HTN?	Elevated	46 (33.2)	28 (65–20)	<0.001
	Stage 1	50.2 (15.6)	50 (50–44)	
	Stage 2	53.2 (20.6)	55 (70–45)	
	Stage 3	37 (25.7)	55 (55–1)	
Medications for T2D	Diet only	49.6 (35.6)	35 (98–20)	<0.001
	Insulin	36.4 (29.7)	55 (55–1)	
	Tablets	50.4 (17)	50 (58.75–45)	
Medications for HTN	*AB	57.1 (21)	55 (72–45)	<0.001
	*ACEI/ ARB	53.4 (21)	50 (55–45)	
	CCB	52.4 (21)	52.5 (70–45)	
	Diuretic	28.5 (21.1)	20 (44.5–20)	
Dose frequency/day	Once	53.6 (27.3)	55 (75–30)	<0.001
	Twice	45.1 (18.4)	45 (50–44)	
	Thrice	64.3 (7.3)	65 (70–60)	
Any side-effects with the medication	No	53.1 (23)	50 (71.5–40)	<0.001
	Moderate	44.3 (19.4)	45 (55–44)	
	Severe	30.5 (30.7)	25.5 (65–1)	

Mann Whitney-U test was used for variable with two categories.

Kruskal wallis test was used for variables with more than two categories.

*ACEI: Angiotensin converting enzyme inhibitors, ARB: Angiotensin receptor blockers, CCB: Calcium channel blockers

**Table 5:** Multivariable Linear Regression model for measuring the effect of characteristics on VAS and VAS for different diagnoses.

Parameter	Overall		One Disease		Two Diseases		Three Diseases	
	B (S.E)	*p-value*	B (S.E)	*p-value*	B (S.E)	*p-value*	B (S.E)	*p-value*
(Intercept)	65.417 (5.562)	<0.001	29.629 (7.265)	<0.001	-14.217 (4.537)	0.002	50.704 (3.194)	<0.001
Gender = Female	-12.174 (1.511)	<0.001	-23.413 (2.921)	<0.001	11.066 (1.265)	<0.001	-11.271 (3.87)	0.004
Gender = Male	Ref							
Educational level = Higher studies	-3.463 (1.899)	0.068	-11.213 (3.502)	0.001	3.093 (1.436)	0.031	-8.59 (2.263)	<0.001
Educational level = Primary education	6.235 (1.822)	0.001	-34.799 (5.514)	<0.001	4.304 (1.479)	0.004	8.316 (1.387)	<0.001
Educational level = Secondary education	Ref							
Marital status = married	-5.967 (2.898)	0.039	15.804 (4.991)	0.002	41.034 (2.611)	<0.001	-	-
Marital status = unmarried	Ref							
Residence = rural	5.303 (1.48)	<0.001	14.02 (3.452)	<0.001	9.58 (1.274)	<0.001	7.469 (1.489)	<0.001
Residence = urban	Ref							
Occupational activity = Not working	1.74 (1.566)	0.267	-2.513 (3.439)	0.465	0.292 (1.413)	0.836	5.08 (3.172)	0.109
Occupational activity = Working	Ref							
Financial situation satisfaction = Medium	1.823 (1.357)	0.179	-6.428 (2.79)	0.021	17.049 (1.14)	<0.001	9.051 (1.924)	<0.001
Financial situation satisfaction = No	-25.423 (4.101)	<0.001	-44.156 (6.061)	<0.001	-8.185 (3.659)	0.025	-	-
Financial situation satisfaction = Yes	Ref							
Monthly income = >100,000	3.192 (2.654)	0.229	51.185 (7.016)	<0.001	24.619 (1.942)	<0.001	7.709 (2.419)	0.001
Monthly income = 10,000–30,000	13.858 (1.92)	<0.001	13.627 (3.019)	<0.001	-10.898 (2.859)	<0.001	10.839 (1.869)	<0.001
Monthly income = 30,000–60,000	-5.652 (1.648)	0.001	-32.649 (3.216)	<0.001	8.876 (1.46)	<0.001	24.745 (1.969)	<0.001
Monthly income = 60,000–90,0	Ref							
Diagnose = 1	-12.062 (1.997)	-	-	-	-	-	-	-
Diagnose = 2	-8.017 (1.788)	-	-	-	-	-	-	-
Diagnose = 3	Ref							
BMI	0.01 (0.094)	0.916	0.12 (0.135)	0.375	0.003 (0.073)	0.967	-0.132 (0.096)	0.169

A high proportion of comorbid patients were found extremely anxious or depressed. 30% patients had severe problem in pain/ discomfort. 28% patients had moderate problem in their usual activities and 20% patients reported no problem regarding their self-care. Most of the patients were not able to wash or dress themselves. Only few patients had no problem with the dimension of mobility, selfcare, usual activities, pain/discomfort and anxiety/depression (21%, 24%, 25%, 12% and 18% respectively) as shown in Fig 2. Comorbidity had a strong impact on HRQoL of patients.

## Discussion

Obesity significantly increases the risk of T2D and HTN. This is a major contributing reason to the high occurrence of these disorder. HRQoL focuses on the impact of health on a patient’s ability to live a fulfilling life. Anxiety/depression was the most frequently detected problematic EQ-5D-5L dimension in our study, whereas self-care was the least affected. This study also shows that the sociodemographic characteristics of patients affect HRQoL. Lower educational and income levels further decreased the HRQoL of patients in both single and multiple diseases. This result is in accordance with that of previous studies [20]. Diet quality is the single leading predictor of premature death and chronic disease in developing countries[21]. Our data showed that family history of obesity had also negative impact of HRQoL in comorbid patient. It is important to consider this characteristic to assist clinicians in treating such patients. A strong association was found between the patient who recognizes and not recognize their symptom of disease on their HRQoL. This might be due to anxiety disorder that could be found easily in such comorbid patients regardless of the treatment regimen [22]. Special attention should be given to this population groups within daily clinical practice. We explored that hospitalized patient had negative impact on HRQoL. These findings are compatible to the previous study in which hospitalized patients with multimorbidity experienced more burden of their disease, functional disabilities, and a reduced quality of life [23]. Patients’ subjective feelings about comorbidity were significantly poor, as evidenced by HRQoL VAS ratings. Our results showed improved HRQoL of patients who followed their proper treatment regimen. It was observed that comorbidity (HTN, T2D and obesity) had a strong negative impact on HRQoL compared to the single disease. HRQoL mean scores were quite lower as compared to other studies conducted in Italy and Greece which might be due to the better healthcare facilities and life style in such countries [24,25]. In addition, it is observed that when obesity is accompanied by HTN and T2D, the quality of life worsens. These findings are compatible with another study conducted in Turkey which reported lower mean score of physical component of HRQoL than mental component [26].

Age, as well as lower levels of education and income, will undoubtedly have a negative impact on HRQoL in developing nations like Pakistan. Because of this, nurses in particular shouldn’t ignore these issues when providing care for their patients. Nursing care is essential for improving patients’ HRQoL and reducing disease consequences. According to our study, patients with HTN and obesity had lower mean scores for the physical component of HRQoL. In light of the fact that both HTN and obesity place a greater burden on the patient than the disease itself does, this conclusion appears to be consistent with the literature [27]. Obese and HTN patients require assistance and support while they adjust to their new lifestyle. The patient will be able to comprehend the nature of the disease and manage it by putting the suggested adjustments in lifestyle (taking medications, diet, exercise, stress management) into practice [28]. As a result, they will live longer and experience a higher HRQoL.

This study has several limitations. Firstly, we conducted the study in patients with obesity, HTN and T2D individually and in the form of multimorbidity in various private clinics which might bring selection bias. Secondly, responses of the patients might be subjected to potential bias. Lastly, number of questions might not be enough to determine all the clinical characteristics of the patients.

## Conclusion

Obesity, HTN and T2D resulted in a decrease in the HRQoL. Patients who had one or multiple diseases needed to be supported, accepted and understood, so that they could establish healthy lifestyle, find solutions to their issues, and improve their HRQoL. The regular assessment of patients’ HRQoL should be followed by the identification of their training requirements.

## Supporting information

S1 Checklist (DOC)
